# Women With Complex Vascular Anomalies: Impact on Contraception, Pregnancy and Reproductive Health

**DOI:** 10.1177/12034754241302825

**Published:** 2024-12-14

**Authors:** Jerome Coulombe, Sandra Ondrejchak, Josee Dubois, Julie Powell, Catherine McCuaig, Catherine Farrell, Catherine Taillefer, Elisabeth Rousseau

**Affiliations:** 1 Division of Dermatology, Department of Paediatrics, CHU Ste-Justine, Universite de Montreal, Montreal, QC, Canada; 2 Division of Interventional Radiology, Department of Radiology & Nuclear Medicine, CHU Ste-Justine, Universite de Montreal, Montreal, QC, Canada; 3 Division of Intensive Care Medicine, Department of Paediatrics, CHU Ste-Justine, Universite de Montreal, Montreal, QC, Canada; 4 Department of Obstetrics & Gynecology, CHU Sainte-Justine, Universite de Montreal, Montreal, QC, Canada; 5 Division of Development Health, Department of Paediatrics, CHU Ste-Justine, Universite de Montreal, Montreal, QC, Canada

**Keywords:** obstetrics, gynecology, vascular anomalies, women, pregnancy, and contraception

## Abstract

**Background:**

Literature is scarce regarding contraceptive and reproductive health in women with complex vascular anomalies (VA).

**Objectives:**

To assess obstetrical, gynecological and reproductive health in this population.

**Methods:**

Female adult patients of childbearing age from the VA clinic of a single tertiary care centre in Canada have been recruited by retrospective chart review, and administered phone interviews.

**Results:**

Of the 16 patients recruited (mean age 32.8 years old), 14 used oral contraceptive pills (OCP) and intrauterine devices, with 3 patients experiencing a worsening of their VA under OCP. Fifteen women had children, with 12 experiencing variable worsening of their VA that returned to baseline after delivery or nursing cessation. Three women had severe complications. Degree of complications was variable in each subsequent pregnancy.

**Conclusion:**

Contraception and pregnancy management in women should be individualized and coordinated with a VA referral centre.

## Introduction

Female adult patients with complex vascular anomalies (VA) represent a management challenge for physicians and caregivers. There is a scarcity of data addressing gynecological, obstetrical and reproductive health, especially in women with PIK3CA-related overgrowth syndrome, including Klippel-Trenaunay syndrome (KTS).^1-4^ Our case series aims to evaluate the impacts of VA on contraception, reproductive health, pregnancy histories and complications.

## Method

VA were classified according to the revised ISSVA classification of 2018.^5^ Complex VA patients were selected by retrospective chart review from the multidisciplinary VA clinic of Centre Hospitalier Universitaire Sainte-Justine (CHUSJ). Inclusion criteria were female sex, adult age at the time of the study and a diagnosis of complex VA defined by a history, including 2 or repeated multimodal therapies including sclerotherapy, endovascular embolization, laser therapy and/or surgical resection. Pediatric patients, male patients and VA patients not needing multiple aforementioned therapies were excluded. Patients were contacted by phone by our registered nurse to answer a survey on the impacts of their VA on childbirth, contraception, and obstetrical outcomes. Descriptive statistics were used. Verbal informed consents were obtained by phone. The CHUSJ ethics board approved this study.

## Results

Eighteen adult women were identified by chart review. Sixteen were included; 1 woman was unreachable and the other had died from an arterio-veinous malformation (AVM)-related complication. Median age was 32.8 years old (Supplemental File). Eleven patients had KTS affecting the lower limbs. Fourteen women had used some form of hormonal contraception, oral combined contraceptive pills (OCP) or intrauterine device (IUD) in the past. Three KTS patients experienced worsening of their VA under OCP, manifesting in increased pain, heaviness and size. Five women eventually opted for definitive surgical options: tubal ligation (1), hysterectomy (1), and male partner vasectomy (3).

Fifteen women had 29 children from 34 pregnancies (Supplemental File). Three women had uneventful pregnancies and deliveries, and 5 spontaneous abortions occurred. Of the 29 living children, 5 were born by c-section, and 9 suffered from mid to late-term prematurity. Twelve women observed an increase in size, heaviness, and pain episodes of their VA during pregnancy, which returned to baseline after deliveries and nursing cessation. Three women had severe VA-related complications during pregnancy or delivery as briefly described below:

### Case 1

A 30-year-old G_0_P_0_A_0_ with possible Parkes-Weber syndrome presenting as multiple AVM fistula of the face, trunk and left cervico-isthmic portion of the uterus bled profusely on her vaginal delivery of a term baby in a small countryside hospital requiring massive blood transfusions in the immediate postpartum period. Her subsequent pregnancies were delivered vaginally after uterine cervix cerclage without further complications. Unfortunately, 10 years later she died from facial AVM complications but she was able to have children with the right monitoring.

### Case 2

A 20-year-old G_1_P_0_A_0_ woman with KTS involving her trunk and right lower limb had an extensive capillaro-veinous malformation encasing her spinal trunk from D_1_ to S_3_. During her third trimester, she developed sudden motor bilateral lower limb paralysis without cauda equina syndrome. MRI showed expansion of her spinal VA. Active intervention was deemed too risky. The woman entered preterm labour at 32 weeks of gestation. C-section delivered a healthy baby and her lower limb motor paralysis improved and disappeared in the next 9 months with diminution of the spinal VA size. In her 2 following pregnancies, she only experienced mild limb paresthesia and had normal vaginal deliveries.

### Case 3

On her prenatal ultrasound, a 20-year-old woman G_1_P_0_A_0_ with left lower limb KTS was found to have numerous uterine and cervical varicosities. She went to have vaginal delivery at term complicated with massive bleeding in the immediate postpartum period that required blood transfusions twice. During her second pregnancy G_2_P_1_A_0_ she suffered from deep venous thrombosis of her left lower leg that required heparin therapy complicated by left knee hemarthrosis. Heparin was discontinued and the baby was delivered vaginally at term without complication.

## Discussion

KTS presents with variable segmental tissue hypertrophy, lymphatic malformations, venous malformations, and dysplastic vessels, that can be associated with localized intravascular coagulation (LIC) ranging from benign to profuse bleeding, deep vein thromboembolism (DVT), pulmonary embolism, or pulmonary hypertension.^1-2,4^ The exact mechanism behind the hypercoagulability in VA is not completely understood but altered blood flow in dysplastic vessels, blood stagnation in ectasic veins and prothrombotic risk factors such as immobilization, OCP and pregnancy are contributive.^3^

In our series, 3 women with KTS experienced worsening of their VA on OCP and switched to an alternate contraceptive method. Expert panels recommend progesterone only or IUDs for contraception in extensive venous VA female patients, especially if signs of LIC are detected when dosing D-Dimers and fibrinogen levels.^4^ Further studies are needed to determine the optimal management of contraception in VA patients.

During pregnancy, only 3 patients reported asymptomatic VA. The majority had variable degrees of VA or obstetrical complications that returned to baseline after delivery or the end of nursing. Three women suffered from severe complications in the peripartum period but not at each pregnancy. In the literature, studies have shown that risk of DVT or bleeding in VA patient pregnancies is comparable with the rate in non-pregnant women with KTS.^2^ Nonetheless, experts agree that peripartum period in complex VA patients is critical and should be carefully planned, monitored, and managed in centres with experience with VA.^1,2^ Premature birth rate in Canada is evaluated at 8%, and stands at 31% in our cohort. However, the heterogeneity of our cohort prevents us to draw any significant explanation for this increased rate.

Our study suffers from its retrospective nature. It is limited by several biases including recruitment in a tertiary care centre of complex VA patients, recall bias, lack of systematic molecular diagnosis, heterogeneous group of VA, and including patients from a pediatric centre now into adulthood. However, it gives insights into the importance of reproductive health counselling in adult women diagnosed with a complex VA in their childhood. Finally, it highlights the importance of transition care coordination in the adult healthcare system to diminish obstetrical complications in this at-risk population to help them have a family if they wish so.

## Supplemental Material

sj-docx-1-cms-10.1177_12034754241302825 – Supplemental material for Women with Complex Vascular Anomalies: Impact on Contraception, Pregnancy and Reproductive Health Supplemental material, sj-docx-1-cms-10.1177_12034754241302825 for Women with Complex Vascular Anomalies: Impact on Contraception, Pregnancy and Reproductive Health by Jerome Coulombe, Sandra Ondrejchak, Josee Dubois, Julie Powell, Catherine McCuaig, Catherine Farrell, Catherine Taillefer and Elisabeth Rousseau in Journal of Cutaneous Medicine and Surgery

sj-docx-2-cms-10.1177_12034754241302825 – Supplemental material for Women with Complex Vascular Anomalies: Impact on Contraception, Pregnancy and Reproductive Health Supplemental material, sj-docx-2-cms-10.1177_12034754241302825 for Women with Complex Vascular Anomalies: Impact on Contraception, Pregnancy and Reproductive Health by Jerome Coulombe, Sandra Ondrejchak, Josee Dubois, Julie Powell, Catherine McCuaig, Catherine Farrell, Catherine Taillefer and Elisabeth Rousseau in Journal of Cutaneous Medicine and Surgery
