# Supplementary material for: Women With Complex Vascular Anomalies: Impact on Contraception, Pregnancy and Reproductive Health
Source: J Cutan Med Surg. 2024 Dec 14;29(2):156–8. doi: 10.1177/12034754241302825 (PMC11979311; doi:10.1177/12034754241302825)
Supplement: sj-docx-1-cms-10.1177_12034754241302825 – Supplemental material for Women with Complex Vascular Anomalies: Impact on Contraception, Pregnancy and Reproductive Health [file sj-docx-1-cms-10.1177_12034754241302825.docx]

Figure S1. Total number of pregnancies in women with complex VA.
