# Supplementary material for: Women With Complex Vascular Anomalies: Impact on Contraception, Pregnancy and Reproductive Health
Source: J Cutan Med Surg. 2024 Dec 14;29(2):156–8. doi: 10.1177/12034754241302825 (PMC11979311; doi:10.1177/12034754241302825)
Supplement: sj-docx-2-cms-10.1177_12034754241302825 – Supplemental material for Women with Complex Vascular Anomalies: Impact on Contraception, Pregnancy and Reproductive Health [file sj-docx-2-cms-10.1177_12034754241302825.docx]

Table S1 Baseline characteristics of the 16 patients with complex VA and reproductive health

| **1- Patients characteristics (%)** | |
| --- | --- |
| Age (years) | 32,8 (23-45) |
| Sex female | 16 (100) |
| Marital status (married) | 13 (81) |
| **2- Vascular anomalie types** | |
| Klippel-Trenaunay syndrome | 13 (82) |
| Arterioveinous | 3 (19) |
| Venous | 1 (1) |
| Lymphatic | 1 (1) |
| **3- Localization** | |
| Lower extremity | 11 (69) |
| Trunk | 5 (31) |
| Head / neck | 3 (18) |
| Upper extremity | 2 (12) |
| **4- Contraception** |  |
| Progesterone only | 10 (63) |
| OCP | 8 (50) |
| Surgery | 5 (31) |
| IUD | 4 (25) |
| Contraceptive vaginal ring | 4 (25) |
| Contraceptive skin patch | 1 (6) |

OCP: oral contraceptive pills, IUD: intrauterine devices
